# Factors Influencing Leaf- and Root-Associated Communities of Bacteria and Fungi Across 33 Plant Orders in a Grassland

**DOI:** 10.3389/fmicb.2019.00241

**Published:** 2019-02-19

**Authors:** Hirokazu Toju, Hiroko Kurokawa, Tanaka Kenta

**Affiliations:** ^1^Center for Ecological Research, Kyoto University, Kyoto, Japan; ^2^Precursory Research for Embryonic Science and Technology, Japan Science and Technology Agency, Kawaguchi, Japan; ^3^Forestry and Forest Products Research Institute, Tsukuba, Japan; ^4^Sugadaira Research Station, Mountain Science Center, University of Tsukuba, Nagano, Japan

**Keywords:** biodiversity, endophytes, host specificity, mycorrhizal fungi, plant-associated microbiomes, plant-soil feedbacks

## Abstract

In terrestrial ecosystems, plants interact with diverse taxonomic groups of bacteria and fungi in the phyllosphere and rhizosphere. Although recent studies based on high-throughput DNA sequencing have drastically increased our understanding of plant-associated microbiomes, we still have limited knowledge of how plant species in a species-rich community differ in their leaf and root microbiome compositions. In a cool-temperate semi-natural grassland in Japan, we compared leaf- and root-associated microbiomes across 137 plant species belonging to 33 plant orders. Based on the whole-microbiome inventory data, we analyzed how sampling season as well as the taxonomy, nativeness (native or alien), lifeform (herbaceous or woody), and mycorrhizal type of host plants could contribute to variation in microbiome compositions among co-occurring plant species. The data also allowed us to explore prokaryote and fungal lineages showing preferences for specific host characteristics. The list of microbial taxa showing significant host preferences involved those potentially having some impacts on survival, growth, or environmental resistance of host plants. Overall, this study provides a platform for understanding how plant and microbial communities are linked with each other at the ecosystem level.

## Introduction

Plants interact with various taxonomic groups of microbes both in the phyllosphere and rhizosphere (van der Heijden et al., 1998; Berendsen et al., 2012; Bai et al., 2015; Peay et al., 2016). Diverse bacteria and yeasts, for example, are present on leaf surfaces, involved in underappreciated metabolic pathways (Mercier and Lindow, 2000; Delmotte et al., 2009; Hacquard et al., 2015). In addition to those epiphytes, a number of bacteria and filamentous fungi are known to inhabit leaf tissue (Ding and Melcher, 2016; Hamonts et al., 2018), playing pivotal roles in resistance of host plants against biotic and abiotic environmental stresses (Schardl and Phillips, 1997; Arnold et al., 2003; Hardoim et al., 2015; Santoyo et al., 2016). In root systems, mycorrhizal fungi provide plants with soil phosphorus and/or nitrogen, fueling hosts’ growth (Parniske, 2008; Smith and Read, 2008; Tedersoo et al., 2010). Likewise, some endophytic fungal taxa have been known to enhance nutritional conditions of host plants (Newsham, 2011; Hiruma et al., 2016; Almario et al., 2017). Moreover, rhizosphere/endophytic bacteria and fungi associated with roots can increase disease resistance of host plants, possibly by stimulating host immune systems (Ramamoorthy et al., 2001; Weston et al., 2012; Pieterse et al., 2014; Hacquard et al., 2017) or by suppressing soil pathogens directly (Compant et al., 2005; Gao et al., 2010; Durán et al., 2018; Kwak et al., 2018). Thus, understanding of the compositions of plant microbiomes is a prerequisite for understanding the physiology and ecology of plants in terrestrial ecosystems (van der Heijden et al., 2008; Schlaeppi and Bulgarelli, 2015; Toju et al., 2018a).

While exploration of plant microbiomes has been accelerated since the emergence of high-throughput DNA sequencing (Öpik et al., 2009; Lundberg et al., 2012; Bai et al., 2015), we still have limited knowledge of how diverse plant species co-occurring in a grassland or forest ecosystem can differ in their microbiome compositions (Toju et al., 2016a). Moreover, most plant microbiome studies target only bacteria or fungi [but see (Agler et al., 2016)) in either above- or below-ground systems [but see (Bai et al., 2015; Wagner et al., 2016)], precluding comprehensive understanding of microbiome compositions. Given that bacteria and fungi can interact with each other within hosts (Frey-Klett et al., 2007; Hoffman and Arnold, 2010) and that above- and below-ground ecological processes can be interlinked (Bever et al., 2010; Mangan et al., 2010; van der Putten et al., 2013), the targets of plant microbiome studies need to be expanded toward a better understanding of the processes that organize plant and microbial communities in the wild. Studies comparing microbiome compositions across tens (or more) of plant species co-occurring in natural ecosystems (Toju et al., 2014, 2018b), in particular, will allow us to examine what kinds of host properties can contribute to the organization of leaf- and root-associated microbial communities.

In this study, we sampled leaves and roots of 137 plant species representing 111 genera, 55 families, and 33 orders in a cool-temperate grassland in Japan, thereby performing a high-throughput sequencing analysis of both prokaryote and fungal communities associated with plants. The sample set of diverse plant species allowed us to examine what host properties can contribute to variation in leaf and root microbiome compositions in an ecosystem. Furthermore, we statistically tested how each prokaryote or fungal genus showed preferences for seasons as well as preferences for nativeness (native or alien), lifeform (herbaceous or woody), and mycorrhizal type (ectomycorrhizal, arbuscular mycorrhizal, non-mycorrhizal, or variable mycorrhizal) of host plants. Overall, this study, for the first time, shows how more than 100 plant species in a single ecosystem can differ in their leaf and root microbiome compositions depending on their characteristics. The statistical results on plant–microbe associations shed light on underappreciated diversity of host–symbiont associations in grasslands, providing fundamental information for conserving and restoring terrestrial ecosystems.

## Materials and Methods

### Sampling

**FIGURE 1:**
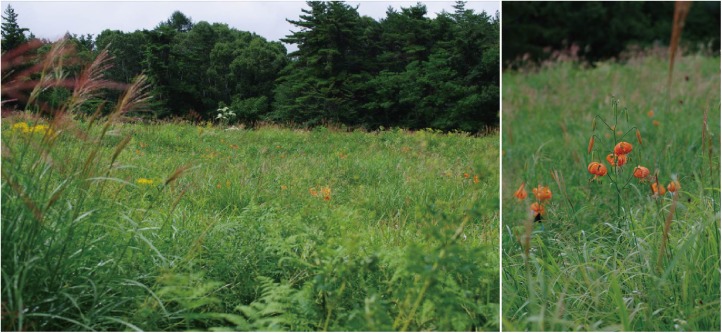
Grassland of Sugadaira Research Station.

Fieldwork was conducted in Sugadaira Research Station, Mountain Science Center, University of Tsukuba, Sugadaira, Ueda, Nagano Prefecture, Japan (36.524°N; 138.349°E; 1340 m asl) (Figure 1). In Sugadaira Research Station, 6 ha of a semi-natural grassland has been maintained by mowing plants in autumn and thereby preventing the community succession to a forest. Thus, woody plant species that occurred in the grassland are shrubs or saplings of tall trees colonized from surrounding forests. In total, 200 plant species have been observed from the grassland, including some alien species non-native to the Japanese Archipelago [plant species uncited in Ohashi et al. (2016)].

In the grassland, both native and alien plant species were sampled to reveal the compositions of prokaryote and fungal communities associated with leaves and roots through summer and autumn (July 19–20, August 16–18, and September 7–8) in 2017. We targeted only non-reproductive plant individuals that had neither flower buds, flowers, nor fruits so that plant physiology and chemistry would not be affected by reproduction [*sensu* Obeso (2002)]. We tried to sample as many plant species as possible within the sampling days in each month. Note that root systems of multiple plant species were tangled with each other at the study site due to the dominance of perennial plants. Therefore, we sampled 1–8 liters of soil (A horizon) including root systems for each target plant individuals and quickly washed the root system in a nearby laboratory to carefully trace root tips directly connected to above-ground tissue of the target plant. A 1-cm^2^ disk of a mature leaf and a 2-cm segment of a terminal root were collected from each plant sample and preserved at -20°C until DNA extraction. After the sampling, remaining plant organs of rare plant species were replaced at the original sampling positions. In total, 289 plant individuals representing 138 plant species (112 genera, 55 families, 33 orders) were collected (Supplementary Data S1). The sampling points were scattered across the 6-ha semi-natural grassland of Sugadaira Research Station: sampling in August and September was conducted in the spatial positions that had not been disturbed by sampling in previous months. The fieldwork and research were permitted by Sugadaira Research Station, Mountain Science Center, University of Tsukuba.

### DNA Extraction, PCR, and Sequencing

Each leaf or root sample was surface-sterilized by immersing it in × 1/100 NaClO (Nacalai Tesque) for 1 min and it was subsequently washed in ethanol twice. DNA was extracted with a cetyltrimethylammonium bromide (CTAB) method after pulverizing the roots with 4 mm zirconium balls at 25 Hz for 3 min using a TissueLyser II (Qiagen) (Toju et al., 2013).

For each of the leaf and root samples, the 16S rRNA V4 region of the prokaryotes and the internal transcribed spacer 1 (ITS1) region of fungi were amplified. The PCR of the 16S rRNA region was performed with the forward primer 515f (Caporaso et al., 2011) fused with 3–6-mer Ns for improved Illumina sequencing quality (Lundberg et al., 2013) and the forward Illumina sequencing primer (5′- TCG TCG GCA GCG TCA GAT GTG TAT AAG AGA CAG- [3–6-mer Ns] – [515f] -3′) and the reverse primer 806rB (Apprill et al., 2015) fused with 3–6-mer Ns and the reverse sequencing primer (5′- GTC TCG TGG GCT CGG AGA TGT GTA TAA GAG ACA G [3–6-mer Ns] - [806rB] -3′) (0.2 μM each). To prevent the amplification of mitochondrial and chloroplast 16S rRNA sequences of host plants, specific peptide nucleic acids [mPNA and pPNA; Lundberg et al. (2013)] (0.7 μM each) were added to the reaction mix of KOD FX Neo (Toyobo). The temperature profile of the PCR was 94°C for 2 min, followed by 35 cycles at 98°C (denaturation) for 10 s, 78°C (annealing of PNA) for 10 s, 60°C (annealing of primers) for 30 s, and 68°C (extension) for 50 s, and a final extension at 68°C for 5 min. To prevent generation of chimeric sequences, the ramp rate through the thermal cycles was set to 1°C/sec (Stevens et al., 2013). Illumina sequencing adaptors were then added to respective samples in the supplemental PCR using the forward fusion primers consisting of the P5 Illumina adaptor, 8-mer indexes for sample identification (Hamady et al., 2008) and a partial sequence of the sequencing primer (5′- AAT GAT ACG GCG ACC ACC GAG ATC TAC AC - [8-mer index] - TCG TCG GCA GCG TC -3′) and the reverse fusion primers consisting of the P7 adaptor, 8-mer indexes, and a partial sequence of the sequencing primer (5′- CAA GCA GAA GAC GGC ATA CGA GAT - [8-mer index] - GTC TCG TGG GCT CGG -3′). KOD FX Neo was used with a temperature profile of 94°C for 2 min, followed by 8 cycles at 98°C for 10 s, 55°C for 30 s, and 68°C for 50 s (ramp rate = 1°C/s), and a final extension at 68°C for 5 min. The PCR amplicons of the samples were then pooled after a purification/equalization process with the AMPureXP Kit (Beckman Coulter). Primer dimers, which were shorter than 200 bp, were removed from the pooled library by supplemental purification with AMpureXP: the ratio of AMPureXP reagent to the pooled library was set to 0.6 (v/v) in this process.

The PCR of fungal ITS1 region was performed with the forward primer ITS1F_KYO1 (Toju et al., 2012) fused with 3–6-mer Ns for improved Illumina sequencing quality (Lundberg et al., 2013) and the forward Illumina sequencing primer (5′- TCG TCG GCA GCG TCA GAT GTG TAT AAG AGA CAG- [3–6-mer Ns] – [ITS1F_KYO1] -3′) and the reverse primer ITS2_KYO2 (Toju et al., 2012) fused with 3–6-mer Ns and the reverse sequencing primer (5′- GTC TCG TGG GCT CGG AGA TGT GTA TAA GAG ACA G [3–6-mer Ns] - [ITS2_KYO2] -3′). The buffer and polymerase system of KOD FX Neo was used with a temperature profile of 94°C for 2 min, followed by 35 cycles at 98°C for 10 s, 58°C for 30 s, and 68°C for 50 s, and a final extension at 68°C for 5 min. Illumina sequencing adaptors and 8-mer index sequences were then added in the second PCR as described above. The amplicons were purified and pooled as described above.

The sequencing libraries of the prokaryote 16S and fungal ITS regions were processed in an Illumina MiSeq sequencer (run center: KYOTO-HE; 15% PhiX spike-in). Because the quality of forward sequences is generally higher than that of reverse sequences in Illumina sequencing, we optimized the MiSeq run setting in order to use only forward sequences. Specifically, the run length was set 271 forward (R1) and 31 reverse (R4) cycles to enhance forward sequencing data: the reverse sequences were used only for discriminating between 16S and ITS1 sequences based on the sequences of primer positions.

### Bioinformatics

The raw sequencing data were converted into FASTQ files using the program bcl2fastq 1.8.4 distributed by Illumina. The output FASTQ files were demultiplexed with the program Claident v0.2. 2018.05.29 (Tanabe and Toju, 2013; Tanabe, 2018), by which sequencing reads whose 8-mer index positions included nucleotides with low ( < 30) quality scores were removed. Only forward sequences were used in the following analyses after removing low-quality 3′-ends using Claident. Noisy reads (Tanabe, 2018) were subsequently discarded and then denoised dataset consisting of 2,973,811 16S and 2,774,197 ITS1 reads were obtained. The sequencing data were deposited to DNA Data Bank of Japan (DDBJ^1^) (Accession No.: DRA007062).

For each dataset of 16S and ITS1 regions, filtered reads were clustered with a cut-off sequencing similarity of 97% using the program VSEARCH (Rognes et al., 2014) as implemented in Claident. The operational taxonomic units (OTUs) representing less than 10 sequencing reads were subsequently discarded (Supplementary Data S2). The molecular identification of the remaining OTUs was performed based on the combination of the query-centric auto-*k*-nearest neighbor (QCauto) method (Tanabe and Toju, 2013) and the lowest common ancestor (LCA) algorithm (Huson et al., 2007) as implemented in Claident (Supplementary Data S2). Note that taxonomic identification results based on the combination of the QCauto search and the LCA taxonomic assignment are comparable to, or sometimes more accurate than, those with alternative approaches (Tanabe and Toju, 2013; Toju et al., 2016a; Toju et al., 2016b).

For each combination of target region (16S or ITS1) and sample type (root or soil), we obtained a sample × OTU matrix, in which a cell entry depicted the number of sequencing reads of an OTU in a sample (Supplementary Data S3). The cell entries whose read counts represented less than 0.1% of the total read count of each sample were removed to minimize effects of PCR/sequencing errors (Peay et al., 2015). The filtered matrix was then rarefied to 500 reads per sample using the “rrarefy” function of the vegan 2.5-2 package (Oksanen et al., 2012) of R 3.5.1 (R-Core-Team, 2018). Samples with less than 500 reads were discarded in this process: the numbers of OTUs in the rarefied sample × OTU matrices were 1,470, 5,638, 1,537, and 3,367 for leaf prokaryote, root prokaryote, leaf fungal, and root fungal datasets, respectively (Supplementary Data S4). For each dataset, we also obtained order- and genus-level matrices, which represented order- and genus-level taxonomic compositions of microbes (prokaryotes or fungi), respectively (Supplementary Data S5).

### Prokaryote and Fungal Diversity

Relationships between the number of sequencing reads and that of detected OTUs were examined for respective data matrices (leaf prokaryote, root prokaryote, leaf fungal, and root fungal datasets) with the “rarecurve” function of the R vegan package. Likewise, relationships between the number of samples and that of prokaryote/fungal orders or genera were examined with the vegan “specaccum” function. The order-level taxonomic compositions of leaf prokaryotes, root prokaryotes, leaf fungi, and root fungi were visualized in bar graphs for respective plant orders.

### Factors Contributing to Microbiome Compositions

For each dataset (leaf prokaryote, root prokaryote, leaf fungal, or root fungal dataset), factors contributing to microbial community compositions were examined with the permutational analysis of variance [PERMANOVA; Anderson (2001)] using the vegan “adonis” function (10,000 permutations). Sampling month (July, August, or September) and four variables representing host plant properties were included as explanatory variables. Specifically, order-level plant taxonomy, plant nativeness (native or alien) [based on the list of plant species native to the Japanese Archipelago Ohashi et al. (2016)], plant lifeform (herbaceous or woody), and plant mycorrhizal type [ectomycorrhizal (EcM), arbuscular mycorrhizal (AM), non-mycorrhizal (NM), or variable mycorrhizal (NM-AM)] (Brundrett, 2009) were included as variables representing host properties. In each model, a matrix representing order- or genus-level taxonomic compositions of prokaryotes/fungi was used as the input response matrix. The “Bray-Curtis” metric of *β*-diversity was used in the PERMANOVA analyses.

### Preferences of Each Prokaryote/Fungal Genus

To explore prokaryote/fungal genera that preferentially occurred on plant samples with specific properties, a series of linear regression analyses were performed. For each prokaryote/fungal genus that appeared in 30 or more samples in a genus-level matrix (the leaf prokaryote, root prokaryote, leaf fungal, or root fungal genus-level matrix), a linear regression model of relative abundance (values in a genus-level matrix) was constructed by incorporating a sample property as an explanatory variable. In each model, sampling month (July, August, or September), plant nativeness (native or alien), plant lifeform (herbaceous or woody), or plant mycorrhizal type was examined. By *z*-transforming response variables (i.e., zero-mean and unit-variance), a standardized coefficient was obtained for each combination of a genus and a sample property. Because most plant orders included a few plant species in our datasets, the regression analysis was not applied to plant taxonomy.

## Results

### Prokaryote and Fungal Diversity

**FIGURE 2:**
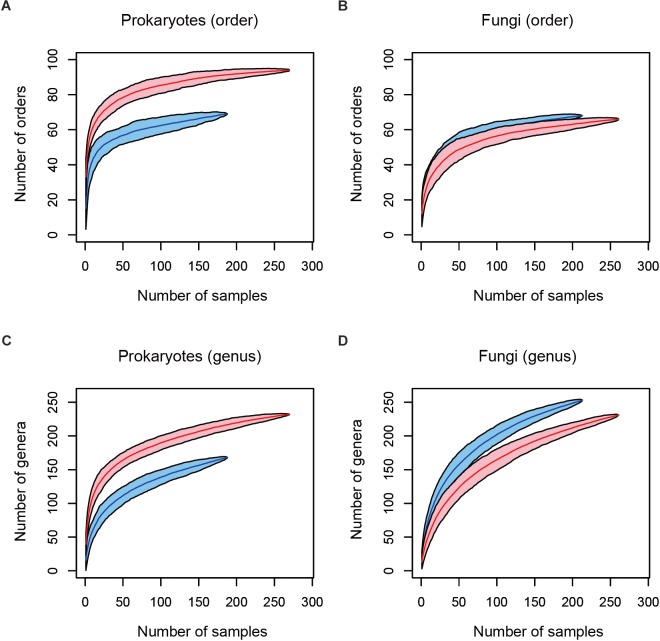
Relationship between the number of leaf/root samples and that of prokaryote/fungal taxa observed. **(A)** Number of prokaryote orders. In total, sequencing data were successfully obtained from 188 leaf and 270 root samples. Blue and red curves represent leaf and root samples, respectively. **(B)** Number of fungal orders. In total, sequencing data were successfully obtained from 213 leaf and 261 root samples. **(C)** Number of prokaryote genera (188 leaf and 270 root samples). **(D)** Number of fungal genera (213 leaf and 261 root samples).

After a series of quality filtering and rarefaction procedures, 41.1 (*SD* = 22.1), 143.4 (*SD* = 37.9), 54.5 (*SD* = 18.8), and 46.0 (*SD* = 22.5) OTUs per sample, on average, were detected, from the leaf prokaryote, root prokaryote, leaf fungal, and root fungal datasets, respectively (Supplementary Figure S1). The number of the samples from which 500 or more reads of prokaryote/fungal sequences were obtained varied among datasets (Figure 2): high proportions of host 16S rRNA and ITS sequences swamped leaf datasets in various taxonomic groups of plants, presumably due to higher concentrations of host organelle/nuclear DNA in leaves than in roots. In total, microbiome data of any of the leaf prokaryote, root prokaryote, leaf fungal, or root fungal community were obtained from 284 plant individuals representing 137 plant species, 111 genera, 55 families, and 33 orders. The numbers of prokaryote orders and genera were higher in root samples than in leaf samples, while those of fungal orders and genera showed opposite patterns (Figure 2).

**FIGURE 3:**
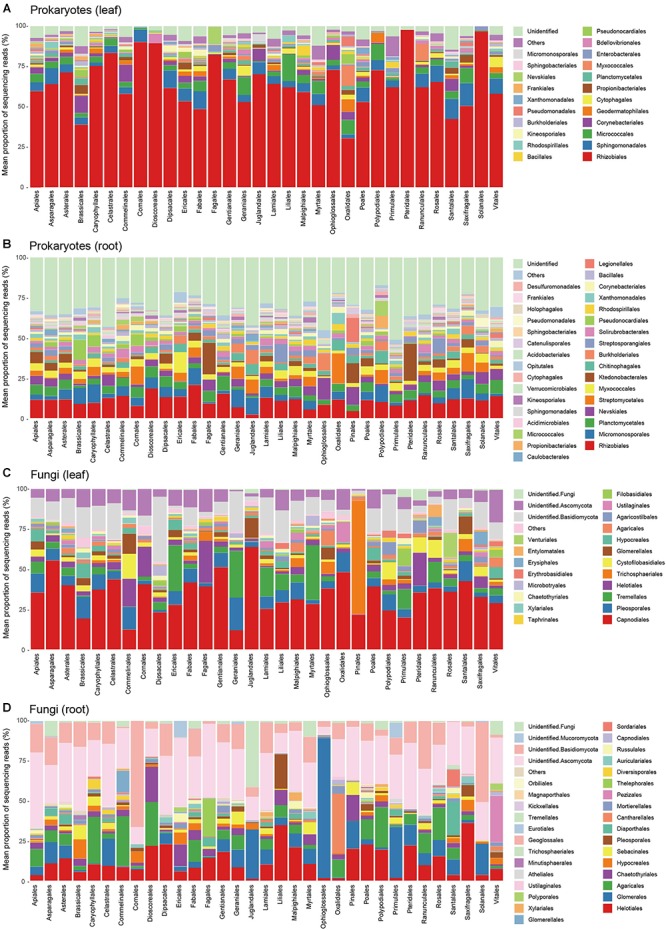
Prokaryote and fungal community compositions. **(A)** Order-level compositions of prokaryotes in leaf samples. Mean proportions of sequencing reads are shown for each plant order. In total, sequencing data were successfully obtained from 188 leaf samples. **(B)** Order-level compositions of prokaryotes in root samples (270 root samples). **(C)** Order-level compositions of fungi in leaf samples (213 leaf samples). Mean proportions of sequencing reads are shown for each plant order. **(D)** Order-level compositions of fungi in root samples (261 root samples).

The leaf prokaryote communities of the examined plants were dominated by the order Rhizobiales, while diverse bacterial taxa constituted the root prokaryote communities (Figure 3A,B). In the leaf fungal communities, the order Capnodiales were the most abundant, while root fungal community compositions varied considerably among host plant orders (Figure 3C,D).

### Factors Contributing to Microbiome Compositions

In the PERMANOVA, sampling month had significant effects on the leaf prokaryote, root prokaryote, and leaf fungal community compositions but not on the root fungal community structure (Table 1 and Supplementary Table S1). Meanwhile, order-level host taxonomy influenced the root prokaryote, leaf fungal, and root fungal community compositions but not the leaf prokaryote community structure (Table 1). The nativeness of host plants (native or alien) had significant impacts on the root prokaryote and the root fungal (genus-level) community compositions (Table 1). The analysis also showed that host plant lifeform (herbaceous or woody) had significant effects on the leaf fungal community structure (Table 1). In contrast, mycorrhizal type of host plants had significant effects on none of the communities examined in the PERMANOVA (Table 1).

**Table 1:** Factors contributing to variation in genus-level community compositions of bacteria and fungi.

Target	Plant tissue	Variable	df	*F*.model	*R*^2^	*P*
Prokaryotes	Leaf	Month	1	3.62	0.019	**0.0070**
		Order	31	1.37	0.218	0.0140
		Native/alien	1	1.34	0.007	0.2159
		Woody/herbaceous	1	0.93	0.005	0.4221
		Mycorrhizal type	3	1.58	0.024	0.0821
	Root	Month	1	3.35	0.011	**0.0005**
		Order	32	1.99	0.217	**0.0001**
		Native/alien	1	2.63	0.009	**0.0041**
		Woody/herbaceous	1	1.32	0.005	0.1824
		Mycorrhizal type	3	0.92	0.009	0.5768
Fungi	Leaf	Month	1	13.35	0.056	**0.0001**
		Order	30	1.63	0.205	**0.0001**
		Native/alien	1	0.95	0.004	0.4333
		Woody/herbaceous	1	5.62	0.024	**0.0001**
		Mycorrhizal type	3	1.34	0.017	0.1338
	Root	Month	1	1.39	0.005	0.1683
		Order	32	1.30	0.158	**0.0075**
		Native/alien	1	3.25	0.012	**0.0015**
		Woody/herbaceous	1	1.37	0.005	0.1826
		Mycorrhizal type	3	1.23	0.014	0.2030

*A PERMANOVA was conducted for each target community (prokaryotes or fungi). The explanatory variables included in the models were sampling month and four host plant properties [order-level taxonomy, nativeness, lifeform (woody or herbaceous), and mycorrhizal type]. P-values significant after a Bonferroni correction are shown in bold for each model (α = 0.05). See Supplementary Table S1 for results on community compositions at the order level.*

### Preferences of Each Prokaryote/Fungal Genus

**FIGURE 4:**
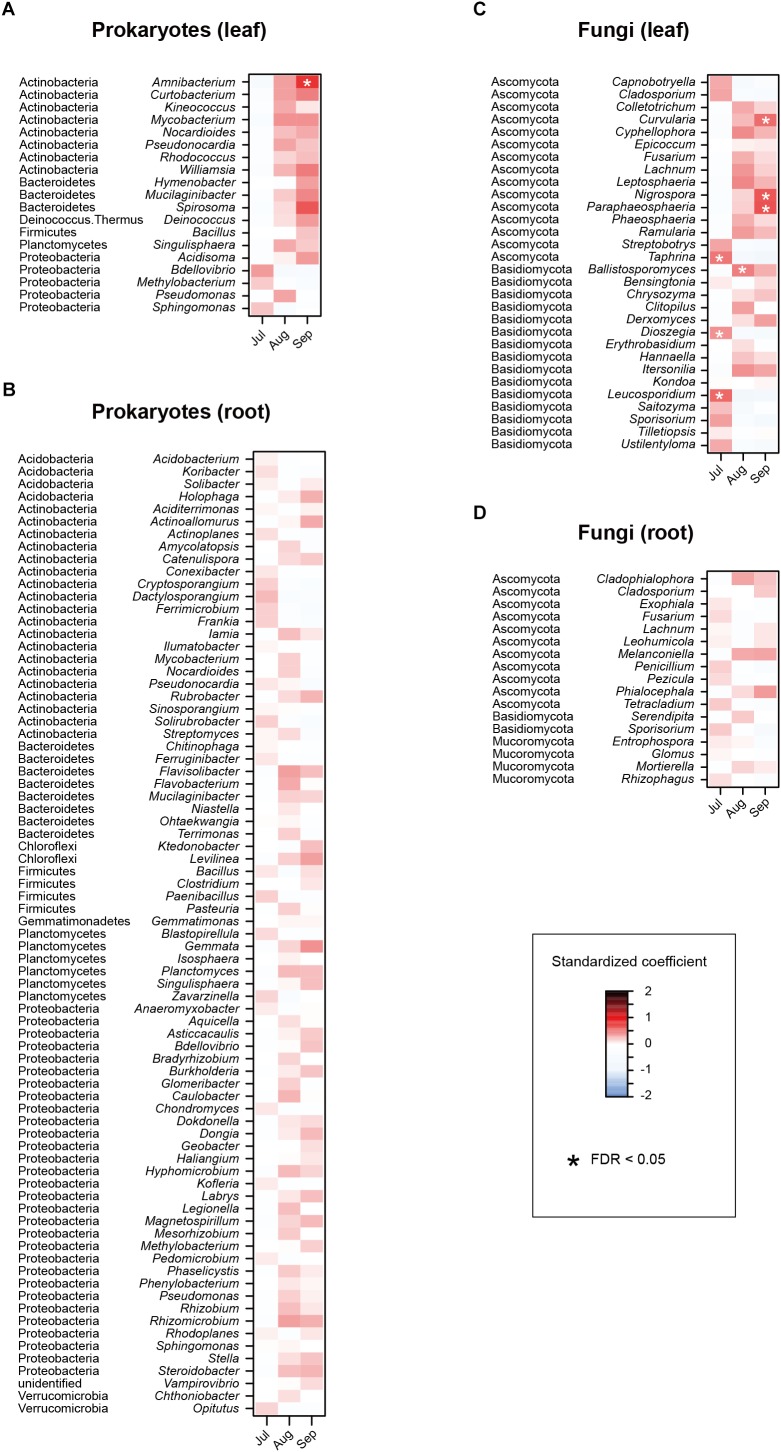
Effects of sampling month on the relative abundances of respective prokaryote and fungal genera. For each prokaryote/fungal genus that appeared in 30 or more samples, a linear model of *z*-standardized (zero-mean and unit-variance) relative abundance was constructed by incorporating sampling month as an explanatory variable. A standardized coefficient was then obtained for each genus × month combination. The *P*-values were converted to false discovery rates (FDRs) by taking into account the number of the genera and months examined. **(A)** Leaf prokaryotes. **(B)** Root prokaryotes. **(C)** Leaf fungi. **(D)** Root fungi.

**FIGURE 5:**
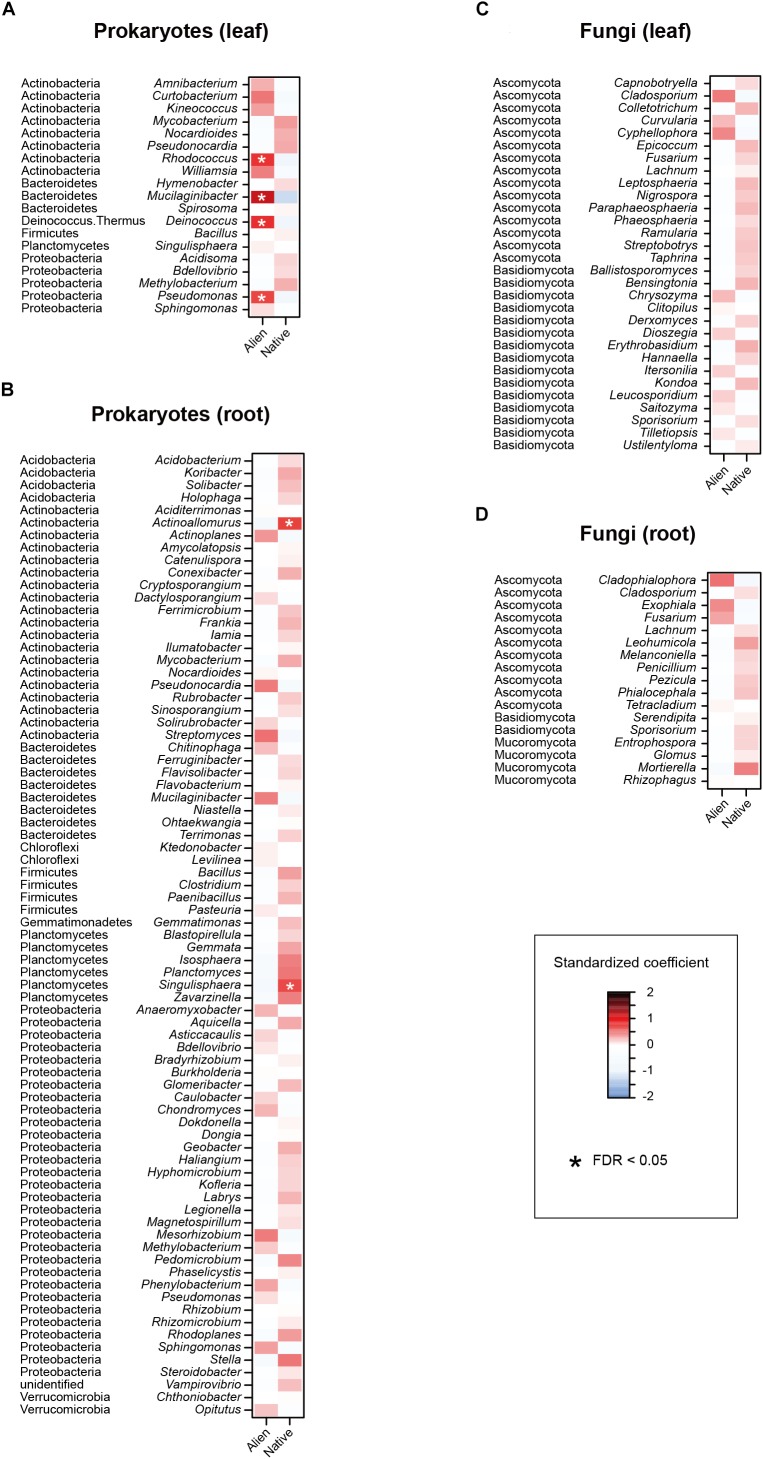
Effects of sampling host plant nativeness on the relative abundances of respective prokaryote and fungal genera. For each prokaryote/fungal genus that appeared in 30 or more samples, a linear model of *z*-standardized (zero-mean and unit-variance) relative abundance was constructed by incorporating host plant nativeness (native/alien) as an explanatory variable. **(A)** Leaf prokaryotes. **(B)** Root prokaryotes. **(C)** Leaf fungi. **(D)** Root fungi.

**FIGURE 6:**
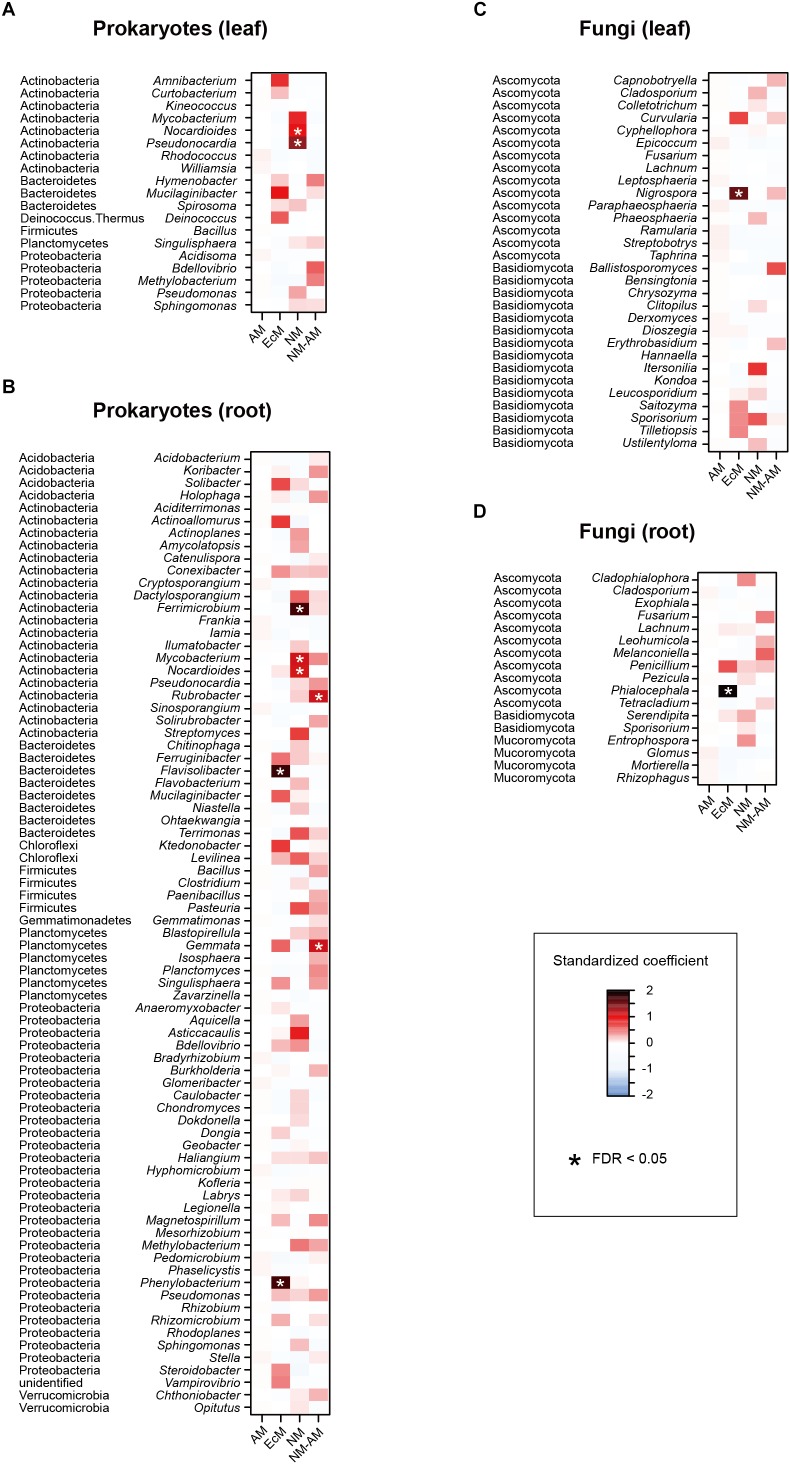
Effects of host-plant mycorrhizal type on the relative abundances of respective prokaryote and fungal genera. For each prokaryote/fungal genus that appeared in 30 or more samples, a linear model of *z*-standardized (zero-mean and unit-variance) relative abundance was constructed by incorporating mycorrhizal type of host plants as an explanatory variable. EcM, ectomycorrhizal; AM, arbuscular mycorrhizal; NM, non-mycorrhizal; NM-AM, variable mycorrhizal (i.e., non-mycorrhizal or arbuscular mycorrhizal). **(A)** Leaf prokaryotes. **(B)** Root prokaryotes. **(C)** Leaf fungi. **(D)** Root fungi.

In the linear regression analyses, the relative abundances of a bacterial genus and eight fungal genera changed through the sampling months in the leaf sample data (Figure 4A,B). For example, the fungal genera *Leucosporidium*, *Taphrina*, and *Dioszegia* in the leaf fungal community appeared preferentially in July, while the bacterial genus *Amnibacterium* preferentially occurred in September (Figure 4). On the other hand, no bacterial/fungal genera showed preferences for sampling months in the root microbiome data (Figure 4C,D). Regarding the nativeness of hosts, bacteria in the genera *Rhodococcus*, *Mucilaginibacter*, *Deinococcus*, and *Pseudomonas* in the leaf microbiome data showed statistically significant preferences for alien plant species, while two leaf-associated bacterial genera, *Actinoallomurus* and *Singulisphaera*, showed preferences for native plant species (Figure 5A,B). No fungal genus showed significant preferences for the nativeness of host plants (Figure 5C,D). Although mycorrhizal type of host plants did not have significant effects in the community-level statistical analysis (Table 1), nine bacterial and two fungal genera showed preferences for host mycorrhizal type (Figure 6). For example, the bacterial genera *Nocardioides* and *Pseudonocardia* preferentially occurred in leaves of non-mycorrhizal plants (Figure 6A). Likewise, three bacterial genera, *Ferrimicrobium*, *Mycobacterium*, and *Nocardioides*, preferentially appeared in the roots of non-mycorrhizal plants (Figure 6B). Meanwhile, *Flavisolibacter* and *Phenylobacterium* showed preferences for ectomycorrhizal plant roots, while Rubrobacter and Gemmata displayed preferences for variable mycorrhizal (NM-AM) plants (Figure 6B). In the fungal community, *Nigrospora* in the leaf data and *Phialocephala* in the root data occurred preferentially in ectomycorrhizal plant samples (Figure 6C,D). Among the prokaryote and fungal genera examined, none showed statistically significant preferences for the lifeform of host plants (Supplementary Figure S2).

## Discussion

Based on a high-throughput sequencing dataset, we herein compared leaf and root microbiome compositions across co-occurring plant species in an ecosystem. By targeting a plant-species-rich grassland in the cool-temperate climate, we compared leaf- and root-associated microbial communities across 33 plant orders (Figure 3) and then performed a series of statistical analyses on factors that may influence community compositions of plant-associated microbes (Figure 4–6 and Table 1). Hereafter, we discuss potential contributions of the factors examined, focusing on preferences of each microbial taxon for host characteristics.

An interesting finding of this study is that, while the compositions of leaf prokaryote, root prokaryote, and leaf fungal communities changed through the sampling months [cf. (Davison et al., 2012; Copeland et al., 2015)], root fungal community compositions did not significantly shift during the period (Table 1). This pattern possibly represents difference in basic environmental features between above- and below-ground systems and/or difference in phenological patterns between prokaryote and fungal communities. For example, above-ground biotic/abiotic environments may be more dynamic than below-ground environments, resulting in rapid turnover of microbial communities. Moreover, above-ground parts of plants are more likely to be accessed by wind-dispersed spores and inocula than below-ground parts (Berg et al., 2014): hence, above-ground microbiome processes may be susceptible to continual immigration. In addition to potential contrasting features of above- vs. below-ground systems, difference in basic ecology between bacteria and fungi may have contributed to the varied phenological patterns. While mycorrhizal and endophytic fungi usually persist on/around host root systems in the form of hyphal networks (Lian et al., 2006; Smith and Read, 2008; Hiruma et al., 2016; Almario et al., 2017), bacterial communities may consist mainly of opportunistic symbionts [*sensu* (Hardoim et al., 2008)], which undergo rapid population growth under favorable environmental conditions and are subsequently replaced by others. Year-round comparative studies on leaf and root microbiomes are awaited for gaining more comprehensive understandings of microbiome dynamics.

Among the microbial communities examined, both root-associated prokaryote and fungal communities significantly varied between native and alien plant species (Table 1). Meanwhile, a series of regression analyses targeting respective microbial genera indicated that some bacterial genera occurred preferentially in the leaves or roots of sampled plants (Figure 5A,B). Among the genera showing preferences for alien plant leaves, *Rhodococcus* is known to involve plant pathogens (Vereecke et al., 2000), while *Mucilaginibacter* includes species with xylan- and pectin-degrading abilities (Pankratov et al., 2007) (Figure 5A). *Singulisphaera*, which showed preferences for native plant roots (Figure 5B), is a genus of bacteria reported from cold, acidic environments (Kulichevskaya et al., 2008). The analysis also showed that some genera in the phylum Actinobacteria (*Rhodococcus* and *Actinoallomurus*) showed preferences for native or alien plant species. Given that many actinomycete bacteria produce chemicals suppressing other microbes (Qin et al., 2011; Bérdy, 2012), their ecological roles in ecosystems are of particular interest. Overall, these results suggest that various taxonomic groups of bacteria are preferentially associated with native or alien plant species, potentially affecting invasiveness of alien plants both positively and negatively (Mitchell and Power, 2003; Moora et al., 2011; Majewska et al., 2015).

At the whole community level, mycorrhizal types of host plants did not have significant effects on plant microbiome compositions (Table 1), unlike previous studies comparing root-associated microbial communities between co-occurring plants with different mycorrhizal types (Toju et al., 2016a; Toju and Sato, 2018). Nonetheless, the analyses for respective microbial taxa highlighted diverse bacterial and fungal genera showing statistically significant preferences for host mycorrhizal types (Figure 6). Among the bacteria showing preferences for non-mycorrhizal plants, *Ferrimicrobium* includes species adapted to low pH conditions (Johnson et al., 2009), while the genus *Nocardioides* consists of bacteria described as endophytes (Qin et al., 2012; Han et al., 2013). *Nigrospora*, which occurred preferentially in ectomycorrhizal plant leaves (Figure 6C), is known to include fungi producing chemicals with antiviral and antifungal functions (Kim et al., 2001; He et al., 2012). We also found that possibly endophytic fungi in the genus *Phialocephala* (Fernando and Currah, 1996; Grünig et al., 2008) showed preferences for ectomycorrhizal plants. Thus, the list of microbes preferentially associated with plants with specific mycorrhizal types (Figure 6) sheds new light on potential diversity of bacteria and non-mycorrhizal fungi, whose physiological and ecosystem-scale functions remain to be investigated (Hardoim et al., 2015; Peay et al., 2016).

Although the data collected in this study provide fundamental information of microbial diversity in a grassland ecosystem, the statistical results should be interpreted with caution. For example, it should be acknowledged that the small number of samples per plant species may have affected the comparison of microbiome compositions among plant taxa (Figure 3). The identification of plant roots is time-consuming especially in species-rich grasslands consisting mainly of perennial plants with tangled root systems, limiting the throughput of sampling. Therefore, for more comprehensive profiling of plant microbiomes, we may need to increase the throughput of plant species identification based on molecular taxonomic assignment (i.e., DNA barcoding) of host plants (Hollingsworth et al., 2009; Toju et al., 2013). Another potential pitfall is that the presence of unidentified bacteria and fungi in the dataset may have biased the statistical analyses. Although databases of microbes have been continually updated (Abarenkov et al., 2010), there remain many bacterial and fungal lineages whose taxonomy has not yet been fixed. In particular, below-ground microbiomes are known to involve a number of poorly investigated taxa, whose physiological and ecological functions remain to be uncovered (Buée et al., 2009; Fierer, 2017). Thus, with more reference microbial databases (Langille et al., 2013; Nguyen et al., 2016), we will be able to examine whether the patterns found in the present analysis hold after assigning unidentified OTUs to right categories.

We herein revealed how diverse bacterial and fungal taxa were associated with leaves and roots of the 138 plant species co-occurring in a cool-temperate grassland, focusing on potential contributions of host plant characteristics on microbiome compositions. Although recent ecological studies have highlighted possible feedbacks between plant and microbial community dynamics (Bever et al., 2010; Mangan et al., 2010; van der Putten et al., 2013), we still have limited knowledge of the processes by which species-rich plant communities are maintained by phyllosphere and rhizosphere microbiomes. Accumulating comprehensive inventory data of microbiomes associated with whole plant communities is a prerequisite for advancing our understanding of ecosystem-scale processes. Case studies in various types of terrestrial ecosystems in diverse climatic regions will allow us to elucidate how plant species with different mycorrhizal types often coexist in natural ecosystems (Booth, 2004; Kadowaki et al., 2018) or why some ecosystems are resistant against alien plants, while others are heavily disturbed by invasive species (Mitchell and Power, 2003; Reinhart and Callaway, 2006).

## Author Contributions

HT, HK, and TK designed the work, performed the fieldwork, and wrote the manuscript. HT conducted molecular experiments and analyzed the data.

## Conflict of Interest Statement

The authors declare that the research was conducted in the absence of any commercial or financial relationships that could be construed as a potential conflict of interest.

## Footnotes

1 http://ddbj.nig.ac.jp/DRASearch/
