# Supplementary material for: Factors Influencing Leaf- and Root-Associated Communities of Bacteria and Fungi Across 33 Plant Orders in a Grassland
Source: Front Microbiol. 2019 Feb 19;10:241. doi: 10.3389/fmicb.2019.00241 (PMC6390183; doi:10.3389/fmicb.2019.00241)
Supplement: Supplementary file 1 [file Table_1.pdf]

Supplementary Table 1. Factors contributing to variation in order-level community structures of bacteria and fungi.

Target	Plant tissue	Variable	df	*F*.model	R^2	*P*
Prokaryotes	Leaf	Month	1	3.65	0.020	**0.0063**
		Order	31	1.08	0.180	0.2952
		Native/alien	1	2.08	0.011	0.0641
		Woody/herbaceous	1	0.73	0.004	0.6182
		Mycorrhizal type	3	1.25	0.020	0.2083
	Root	Month	1	3.06	0.010	**0.0009**
		Order	32	2.17	0.232	**0.0001**
		Native/alien	1	3.30	0.011	**0.0006**
		Woody/herbaceous	1	1.05	0.004	0.3737
		Mycorrhizal type	3	0.79	0.008	0.8083
Fungi	Leaf	Month	1	10.35	0.043	**0.0001**
		Order	30	1.82	0.228	**0.0001**
		Native/alien	1	0.60	0.002	0.8019
		Woody/herbaceous	1	3.39	0.014	**0.0014**
		Mycorrhizal type	3	1.73	0.022	0.0249
	Root	Month	1	1.28	0.005	0.2329
		Order	32	1.48	0.179	**0.0001**
		Native/alien	1	1.03	0.004	0.3946
		Woody/herbaceous	1	0.69	0.003	0.7104
		Mycorrhizal type	3	0.89	0.010	0.6118

A PERMANOVA was conducted for each target community (prokaryotes or fungi). The explanatory variables included in the models were sampling month and four host plant properties [order-level taxonomy, nativeness, life style (woody or herbaceous), and mycorrhizal type]. *P* values significant after a Bonferroni correction are shown in bold for each model ($\alpha = 0.05$).
