# Supplementary figures and images for: Factors Influencing Leaf- and Root-Associated Communities of Bacteria and Fungi Across 33 Plant Orders in a Grassland
Source: Front Microbiol. 2019 Feb 19;10:241. doi: 10.3389/fmicb.2019.00241 (PMC6390183; doi:10.3389/fmicb.2019.00241)

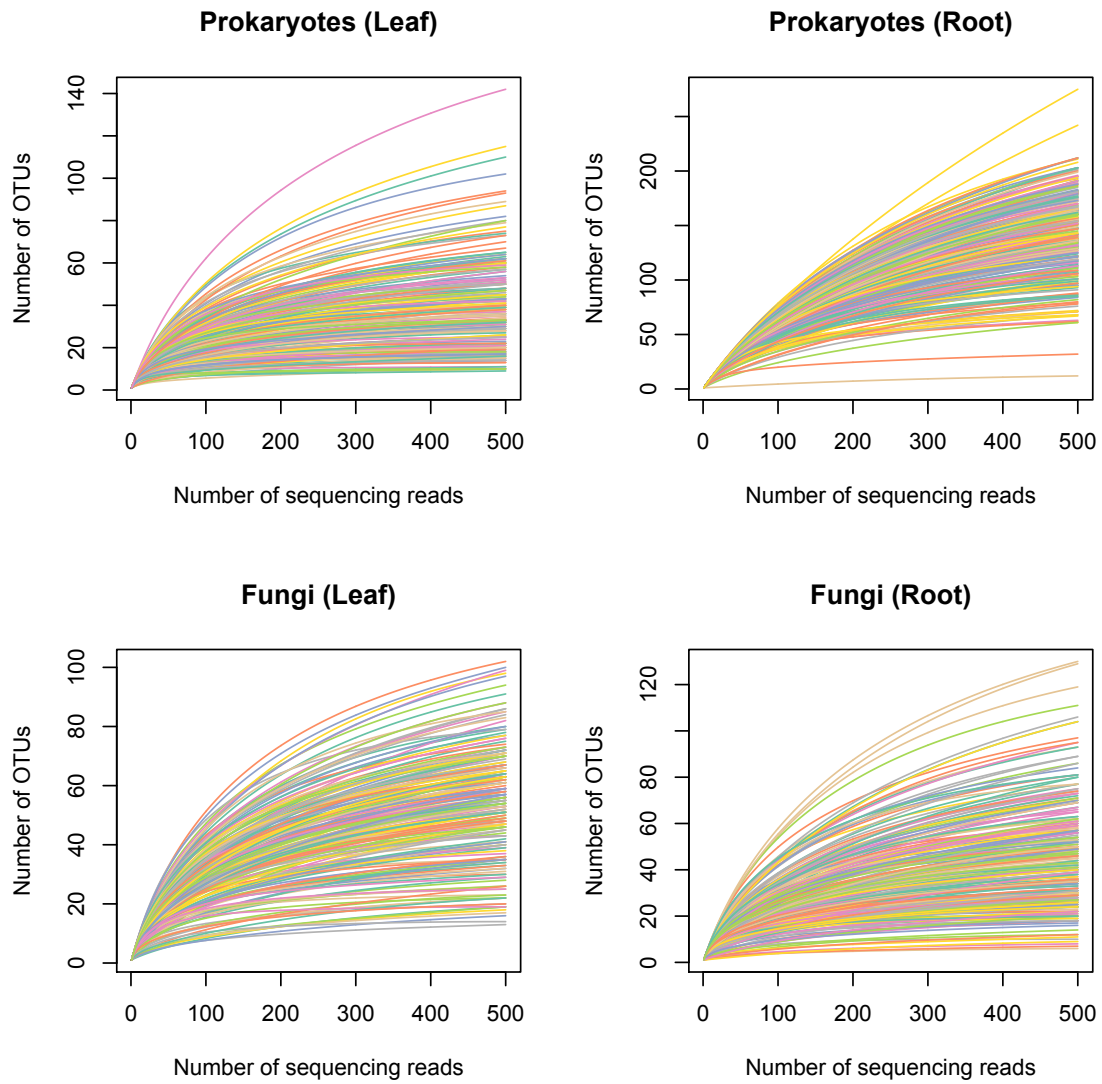

**Supplementary Figure 1.** Relationships between the number of sequencing reads and that of OTUs.

Supplement: Supplementary file 7 [file Image_1.pdf]
