# Supplementary material for: Factors Influencing Leaf- and Root-Associated Communities of Bacteria and Fungi Across 33 Plant Orders in a Grassland
Source: Front Microbiol. 2019 Feb 19;10:241. doi: 10.3389/fmicb.2019.00241 (PMC6390183; doi:10.3389/fmicb.2019.00241)
Supplement: Supplementary file 8 [file Image_2.pdf]

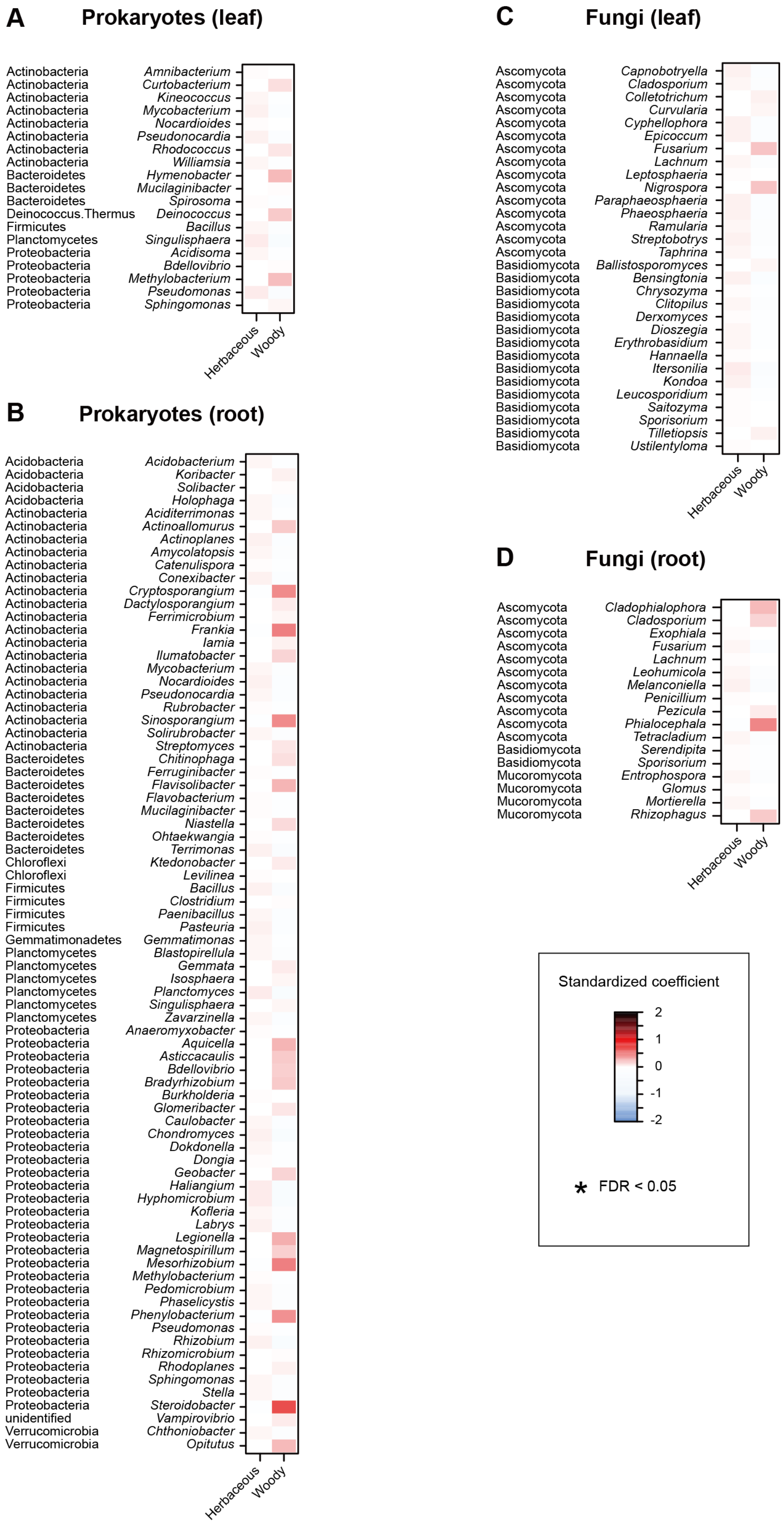
A
Prokaryotes (leaf)
Actinobacteria Amnibacterium
Actinobacteria Curtobacterium
Actinobacteria Kineococcus
Actinobacteria Mycobacterium
Actinobacteria Nocardioides
Actinobacteria Pseudonocardia
Actinobacteria Rhodococcus
Actinobacteria Williamsia
Bacteroidetes Hymenobacter
Bacteroidetes Mucilaginibacter
Bacteroidetes Spirosoma
Deinococcus.Thermus Deinococcus
Firmicutes Bacillus
Planctomycetes Singulisphaera
Proteobacteria Acidisoma
Proteobacteria Bdellovibrio
Proteobacteria Methylobacterium
Proteobacteria Pseudomonas
Proteobacteria Sphingomonas
Herbaceous
Woody
B
Prokaryotes (root)
Acidobacteria Acidobacterium
Acidobacteria Koribacter
Acidobacteria Solibacter
Acidobacteria Holophaga
Actinobacteria Aciditerrimonas
Actinobacteria Actinoallomurus
Actinobacteria Actinoplanes
Actinobacteria Amycolatopsis
Actinobacteria Catenulispora
Actinobacteria Conexibacter
Actinobacteria Cryptosporangium
Actinobacteria Dactylosporangium
Actinobacteria Ferrimicrobium
Actinobacteria Frankia
Actinobacteria Iamia
Actinobacteria Ilumatobacter
Actinobacteria Mycobacterium
Actinobacteria Nocardioides
Actinobacteria Pseudonocardia
Actinobacteria Rubrobacter
Actinobacteria Sinosporangium
Actinobacteria Solirubrobacter
Actinobacteria Streptomyces
Bacteroidetes Chitinophaga
Bacteroidetes Ferruginibacter
Bacteroidetes Flavisolibacter
Bacteroidetes Flavobacterium
Bacteroidetes Mucilaginibacter
Bacteroidetes Niastella
Bacteroidetes Ohtaekwangia
Bacteroidetes Terrimonas
Chloroflexi Ktedonobacter
Chloroflexi Levilinea
Firmicutes Bacillus
Firmicutes Clostridium
Firmicutes Paenibacillus
Firmicutes Pasteuria
Gemmatimonadetes Gemmatimonas
Planctomycetes Blastopirellula
Planctomycetes Gemmata
Planctomycetes Isosphaera
Planctomycetes Planctomyces
Planctomycetes Singulisphaera
Planctomycetes Zavarzinella
Proteobacteria Anaeromyxobacter
Proteobacteria Aquicella
Proteobacteria Asticcacaulis
Proteobacteria Bdellovibrio
Proteobacteria Bradyrhizobium
Proteobacteria Burkholderia
Proteobacteria Glomeribacter
Proteobacteria Caulobacter
Proteobacteria Chondromyces
Proteobacteria Dokdonella
Proteobacteria Dongia
Proteobacteria Geobacter
Proteobacteria Haliangium
Proteobacteria Hyphomicrobium
Proteobacteria Kofleria
Proteobacteria Labrys
Proteobacteria Legionella
Proteobacteria Magnetospirillum
Proteobacteria Mesorhizobium
Proteobacteria Methylobacterium
Proteobacteria Pedomicrobium
Proteobacteria Phaselicystis
Proteobacteria Phenylobacterium
Proteobacteria Pseudomonas
Proteobacteria Rhizobium
Proteobacteria Rhizomicrobium
Proteobacteria Rhodoplanes
Proteobacteria Sphingomonas
Proteobacteria Stella
Proteobacteria Steroidobacter
unidentified Vampirovibrio
Verrucomicrobia Chthoniobacter
Verrucomicrobia Opitutus
Herbaceous
Woody
C
Fungi (leaf)
Ascomycota Capnobotryella
Ascomycota Cladosporium
Ascomycota Colletotrichum
Ascomycota Curvularia
Ascomycota Cyphellophora
Ascomycota Epicoccum
Ascomycota Fusarium
Ascomycota Lachnum
Ascomycota Leptosphaeria
Ascomycota Nigrospora
Ascomycota Paraphaeosphaeria
Ascomycota Phaeosphaeria
Ascomycota Ramularia
Ascomycota Streptobotrys
Ascomycota Taphrina
Basidiomycota Ballistosporomyces
Basidiomycota Bensingtonia
Basidiomycota Chrysozyma
Basidiomycota Clitopilus
Basidiomycota Derxomyces
Basidiomycota Dioszegia
Basidiomycota Erythrobasidium
Basidiomycota Hannaella
Basidiomycota Itersonilia
Basidiomycota Kondoa
Basidiomycota Leucosporidium
Basidiomycota Saitozyma
Basidiomycota Sporisorium
Basidiomycota Tilletiopsis
Basidiomycota Ustilentyloma
Herbaceous
Woody
D
Fungi (root)
Ascomycota Cladophialophora
Ascomycota Cladosporium
Ascomycota Exophiala
Ascomycota Fusarium
Ascomycota Lachnum
Ascomycota Leohumicola
Ascomycota Melanconiella
Ascomycota Penicillium
Ascomycota Pezicula
Ascomycota Phialocephala
Ascomycota Tetracladium
Basidiomycota Serendipita
Basidiomycota Sporisorium
Mucoromycota Entrophospora
Mucoromycota Glomus
Mucoromycota Mortierella
Mucoromycota Rhizophagus
Herbaceous
Woody
Standardized coefficient
2
1
0
-1
-2
* FDR < 0.05

Supplementary Figure 2. Effects of sampling host plant lifeform on the relative abundances of respective prokaryote and fungal genera. For each prokaryote/fungal genus that appeared in 30 or more samples, a linear model of *z*-standardized (zero-mean and unit-variance) relative abundance was constructed by incorporating host plant lifeform (woody or herbaceous) as an explanatory variable. **(A)** Leaf prokaryotes. **(B)** Root prokaryotes. **(C)** Leaf fungi. **(D)** Root fungi.
